# A novel Energy Resources Allocation Management model for air pollution reduction

**DOI:** 10.3389/fpubh.2022.1035395

**Published:** 2023-01-06

**Authors:** Armita Khorsandi, Liping Li

**Affiliations:** ^1^Injury Prevention Research Centre, Shantou University Medical College, Shantou, China; ^2^School of Public Health, Shantou University, Shantou, China

**Keywords:** air pollution reduction, simulation-optimization model, Energy Resources Allocation Management (ERAM), particulate matter (PM), Tehran

## Abstract

Although air pollution has been reduced in various industrial and crowded cities during the COVID-19 pandemic, curbing the high concentration of the crisis of air pollution in the megacity of Tehran is still a challenging issue. Thus, identifying the major factors that play significant roles in increasing contaminant concentration is vital. This study aimed to propose a mathematical model to reduce air pollution in a way that does not require citizen participation, limitation on energy usage, alternative energies, any policies on fuel-burn style, extra cost, or time to ensure that consumers have access to energy adequately. In this study, we proposed a novel framework, denoted as the Energy Resources Allocation Management (ERAM) model, to reduce air pollution. The ERAM is designed to optimize the allocation of various energies to the recipients. To do so, the ERAM model is simulated based on the magnitude of fuel demand consumption, the rate of air pollution emission generated by each energy per unit per consumer, and the air pollution contribution produced by each user. To evaluate the reflectiveness and illustrate the feasibility of the model, a real-world case study, i.e., Tehran, was employed. The air pollution emission factors in Tehran territory were identified by considering both mobile sources, e.g., motorcycles, cars, and heavy-duty vehicles, and stationary sources, e.g., energy conversion stations, industries, and household and commercial sectors, which are the main contributors to particulate matter and nitrogen dioxide. An elaborate view of the results indicates that the ERAM model on fuel distribution could remarkably reduce Tehran's air pollution concentration by up to 14%.

## 1. Introduction

Air pollution, which is inevitable by the rapid growth of technology, is a major problem worldwide. Ambient air pollution that harms living organisms not only threatens the health and longevity of humans and animals but also involves the entire environment. Manifold causes of air pollution made by human activities can be named fossil fuel burning, vehicular, agricultural activities, factories' and industries' pollutant emissions, and indoor and outdoor activities. Natural factors included wildfires, extreme weather events, and volcanic emissions. These create a considerable amount of carbon monoxide and dioxide, nitrogen oxide, volatile organic compounds, surfer dioxides, ozone, and particulates in the air. All these particles spread in our lungs and body and make diseases such as respiratory diseases and asthma, cardiovascular diseases and stroke, fetal growth and child health problems, neuropsychiatric complications, or even cancer (1, 2). The disastrous destruction problems for ecosystems and plants are noted as global warming, acid rain, climate change problem, habitat destruction, the extinction of wildlife, depletion of the ozone layering, temperature inversion due to the emissions of greenhouse gasses to the atmosphere, and other significant environmental impacts of air pollution (3, 4).

There is an assessment that 92% of the globe's people are living in an area where the air quality is not under the current World Health Organization (WHO)'s air pollutants index value approximately (5). About 4.3 million people died from household air pollution and 3.7 million from ambient air pollution, most of whom (3.3 and 2.6 million annually, respectively) lived in Asia (6). Outdoor air pollution stands among the top five life-threatening factors worldwide, mostly in low- and middle-economic countries with almost 4 million deaths in 2016, according to the Global Burden of Disease 1990–2016 research (7). A previous study was conducted on the positive effects of electric and hybrid vehicles on reducing air pollution (8). The study by Ku (9) showed a correlation between land use patterns and air pollutant concentrations due to urbanization, which led to increased energy consumption and the massive emission of air pollutants. Research in the United States (10) revealed that short-term exposure to air pollution, especially to particulate matter, may contribute to the spread and course of the pandemic. A study in China reported that type 2 diabetes mellitus mortality was positively related to short-term exposure to air pollutants during 2013–2019 (11).

Research on the megacity of Tehran indicated that the average particulate matter concentration would increase by 30% in the following decades (12). An investigation showed that, in addition to anthropogenic activity contributions, the widespread drought in Iran and decreasing annual airborne asbestos fibers had caused a significant increase in natural dust in total particulate matter mass (13). Studies demonstrated that exposure to particulate matter has the highest health impact on the residents of Tehran and increments in total mortality by up to 4.6%. Sulfur dioxide, nitrogen dioxide, and ozone stand on the next steps causing almost 3, 2.2, and 1.7% of total mortality in 1 year in 2012, respectively (14). In a study by Vafa-Arani et al. (15), a dynamic system was simulated under several scenarios containing the transportation and industrial sectors' data history in Tehran. Outcomes showed the effectiveness of the technology improvement in fuel, the automotive industry, and the development of public transportation infrastructure policies. Researchers found that more than 90% of the CO gas is generated by transportation in Tehran (16). Using electric motorcycles instead of gasoline-fueled motorcycles has been recommended by the Municipality of Tehran. According to eco-indicators (17), e-motorbikes and conventional bikes have the lowest impact on the environment, and while they cost two or three times more than standard gasoline motorcycles, we cannot expect all the owners to buy e-vehicle instead of their current carburetor motorcycles (18).

The study (19) surveyed the efficiency of their measures to decrease particulate matter and nitrogen dioxide concentration related to the traffic, industrial, and residential combustion sectors in Porto, using The Air Pollution Model based on the 3-D Eulerian model. In (20), the Danish Eulerian Hemispheric Model was considered for air pollution intercontinental transport between North America and Europe. With the help of the multi-objective linear programming problem, a minimization problem on total costs, lead time, and carbon monoxide emissions has been simulated (21). Although there exist many well-examined works in the literature on the topic of air pollution studies, there are very few studies that have utilized a practical mathematical model to address feasible air pollution reduction from various perspectives. Most research benchmarks in the air pollution field are about the dangers of air pollution on human health and mortality risk (22) or need citizen participation and some changing individual behavior to abate air pollution (23). However, our method is based on a precise optimal model that is logically applied and does not emphasize amending people's energy-consuming style to reduce air pollution.

In this research, we proposed a simulation-optimization method to detract the air pollution that does not require changes in the amount of consumers' energy consumption trend, waning and optimizing the demand, or extra costs, which consequently reduces the mortality risk associated with air pollution. Specifically, an optimization model, namely, Energy Resources Allocation Management (ERAM) model, is proposed to have optimal management of allocating various energies to the consumers. The efficiency of the ERAM model for the air pollution scale is higher because of making decisions based on the distribution of various energy resources among the different sectors. This model is new, accurate, and optimal and achieves the highest possible level of air quality. The ERAM model receives the amount of consumed energy requirement, the rate of pollution produced per energy per user, and the total pollution generated by each consumer, as the input, to optimally assign energies to the customers. In summary, the main contribution of this study is as follows:

1. Proposing an integrated simulation-optimization model to optimize the energy resource allocation among different recipient sectors for air pollution mitigation, which is precisely simulated under a decision-making tolerance value.
2. Setting up a real-world case study for evaluating the influence and performance of the compatible proposed method on air pollution reduction without the need for energy consumption reduction.
3. This method applies to every region, and the proposed model can simulate the air pollution problem in each area according to the conditions.

The rest of this study is organized as follows. Section 2 reviews the six principal air pollutants mentioned by the WHO and introduces the optimization model. Section 3 presents the methodology and the details of the ERAM model. In Section 4, the real-world case study is prepared to illustrate the validity and feasibility of the proposed method. A comparative analysis is presented in Section 5 to figure out the priority of our model over previous models. Eventually, we draw our discussion and conclusions in Sections 6 and 7, respectively.

## 2. Preliminaries

### 2.1. Air major pollutants

According to the WHO standards, particulate matter (PM_10_ and PM_2.5_), ozone (O_3_), nitrogen dioxide (NO_2_), carbon monoxide (CO), sulfur dioxide (SO_2_), and lead are the six main elements of air pollution that harm human health and the ecosystem.

### 2.2. The framework of the optimization model

Nowadays, mathematical modeling is the most powerful tool for simulating problems, and it has inevitable aspects in many fields such as economics, management, network, and power engineering (24, 25). Optimization models have been created for optimizing the objective function with respect to the constraints. Optimization models are a portion of mathematical models that can be classified into two general categories: (I) linear programming (LP) and (II) geometric programming (GP) problems. Here, the formulation of the linear programming model is presented. The optimization model consists of two parts: the objective function and the constraint function. The objective function simulates based on the maximization or minimization of the target. The aim of the problem creates the objective function of the optimization model. Constraint functions simulate based on all the problem's limitations.

**Definition 1**. The following problem (26):


(1) $$\begin{array}{l} min\;\left(max\right)\;\overset{\tau_{0}}{\underset{j=1}{\sum }}\alpha_{j}\chi_{j}\; \end{array}$$

$$\begin{array}{l} subject\;to\;\overset{\tau_{i}}{\underset{j=1}{\sum }}\beta_{ij}\chi_{j}\geq \;t_{i},\;\left(1\leq i\leq n^{{}^{\prime }}\right),\\ \;\overset{\tau_{i}}{\underset{j=1}{\sum }}\beta_{ij}\chi_{j}\leq \;l_{i},\;\left(n^{{}^{\prime }}+1\leq i\leq n\right),\\ \;{\chi_{j}}^{-}\leq \chi_{j}\leq {\chi_{j}}^{+},\\ \;\chi \geq 0, \end{array}$$

is called a posynomial linear programming problem of χ, where $\;\chi ={\left(\chi_{1},\;\ldots ,\;\chi_{\tau_{i}}\right)}^{T}$ is a τ_*i*_-dimensional decision variable vector and *T* represents a transpose symbol. In addition, coefficients α_*j*_>0 and β_*ij*_>0 are constant real numbers. *t*_*i*_, *l*_*i*_, ${\chi_{j}}^{-}$, and ${\chi_{j}}^{+}$ are right-hand side, lower and upper bound arbitrary non-negative decision vectors, respectively.

## 3. Material and methods

### 3.1. Methodology

**Figure 1:**
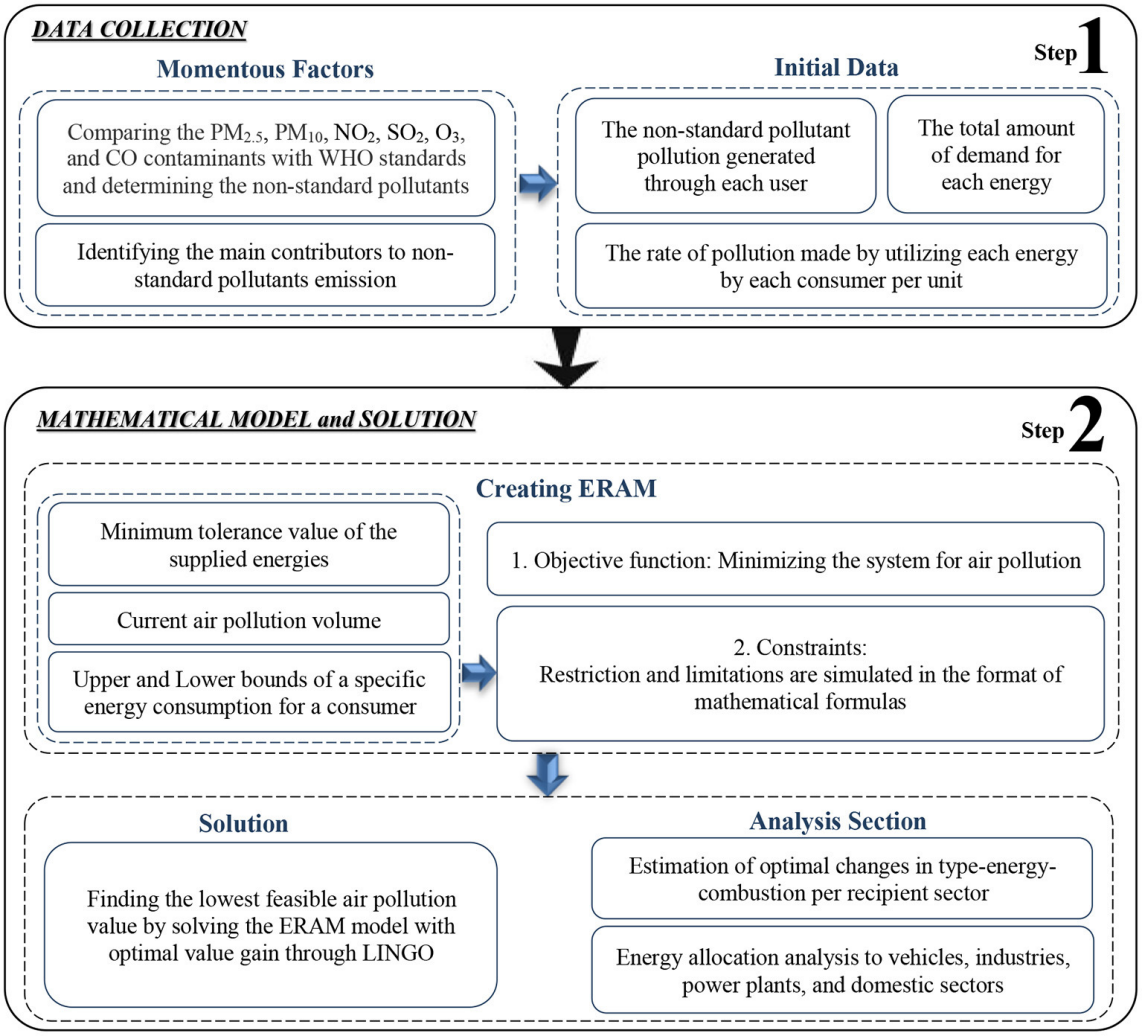
The overall framework of the method for detecting optimal air quality.

The presented method can be classified into two general steps, including data collection and the optimization model. The first step has focused on identifying the major air pollutants greater than the WHO air quality standards. For this issue, the contributors that generate air pollution-related to non-standard pollutants were recognized. The concerned data on the volume of air pollution made through each contributor, the rate of pollution generated through each energy by the consumer usage per unit, and the amount of the consumer's demand for each energy was collected. The second step is assigned to the simulation of the problem with the novel precise optimization model, ERAM, using the dataset collected in step 1. The proposed method is summarized in Figure 1.

*Step 1. Data collection:* This step is divided into two parts. The first part is related to air pollutants. First, the six air pollutants of the study area were compared with the WHO air quality standards. Then, for those air contaminants higher than the standards, the contributors must be distinguished. The second part is concerned with the dataset. Compiling a comprehensive database is required to collect three sets of data based on the determined non-standard pollutants. The first set of data is concerned with the mass of air pollution of the non-standard pollutants, which is generated by each consumer individually. The second group is concerned with energy demand. Here, the energy selling volume in previous years is selected as the lowest amount of energy supply. The selling volume shows that the current quantity of these energies is available. The third set is concerned with the generated pollution contribution by each customer sector in consuming each type of resource energy per unit. The amount of air pollution that a user makes by consuming any energy is different from other users based on its features, and, each energy source makes a special volume of air pollution per unit.

*Step 2. The optimization model:* Since our principal purpose of improvement strategies in the air quality process is the optimal usage of energy resources to reduce air pollution and protect life expectancy, in step 2, an optimization model is proposed. The ERAM programming model is created to operate on total air pollution mitigation, in which the model focuses on minimizing the system. It is expected for the optimal value obtained through the ERAM model to be less than the current air pollution. The collected data in step 1 are employed as the initial data to simulate the objective function of the ERAM model, which minimizes the entire system and the constraints, which are the limitations of the study area. Eventually, the ERAM model is ready to achieve the highest air quality.

Meanwhile, solving the ERAM model gains different categories of feasible solutions; however, only one is the optimal solution. In this approach, different tolerance values make various feasible solution sets. The feasible solutions are all the solutions that can occur through the conditions. All are acceptable but not optimal. The optimal value refers to the best-effected amount on air quality reached by the optimal solution, which indicates the maximum amount achieved in decreasing air pollution. Subsequently, the optimal value indicates the best achievement of the air quality.

*Remark*: Note that none of the factors can be removed or omitted in order to get a better solution. The solution would be to mismanage the allocation. All contributors are active, and the limitations for each user can be changed within the tolerance value of the decision-maker.

### 3.2. Energy Resources Allocation Management model

To perceive how to assign energy resources optimally to users, an appropriate ERAM model is constructed according to the linear programming problem in Eq. 1 as in Eq. 2. Later, it is exclusively depicted how to achieve this formula for simulating the air pollution problem and how to apply it.


(2) $$\begin{array}{l} \min\;\overset{m}{\underset{i=1}{\sum }}\overset{n}{\underset{j=1}{\sum }}r_{ij}\chi_{ij} \end{array}$$

$$\begin{array}{l} s.t.\;\overset{n}{\underset{j=1}{\sum }}r_{ij}\chi_{ij}\geq l_{i},\;\left(1\leq i\leq m\right),\\ \;\overset{m}{\underset{i=1}{\sum }}r_{ij}\chi_{ij}\leq t_{j},\;\left(1\leq j\leq n\right),\\ \chi_{ij}\;\geq {\chi_{ij}}^{-},for\;1\leq i\leq m\;,1\leq j\leq n,\\ \chi_{ij}\;\leq {\chi_{ij}}^{+},for\;1\leq i\leq m\;,1\leq j\leq n,\\ {\chi_{ij}}^{-},\;{\chi_{ij}}^{+}>0,r_{ij}\geq 0,\;\chi_{ij}\geq 0,1\leq i\leq m\;,\;1\leq j\leq n. \end{array}$$

*Objective function*: Since this model intends to diminish the total air pollution, thus, the proposed mathematical model should be a minimization problem. Burning each type of energy by each consumer releases a certain amount of pollutant particles. In this regard, the quantity of particles emitted from each energy burning varies according to the type of user sector. Hence, this specific value constitutes the coefficients relevant to the air pollution distributor sectors, which are the coefficients of the variables in the objective function.

Suppose that *i* is a type of energy used by consumer *j*. The volume of air pollution generates through consuming *i*–th energy by *j*-th user, which can be calculated as Eq. 3.


(3) $$\begin{array}{l} P_{ij}=r_{ij}.\chi_{ij}\;,\;for\;1\leq i\leq m,\;1\leq j\leq n,\; \end{array}$$

where χ_*ij*_ indicates the optimal amount of energy *i*, which is supposed to be devoted to customer *j*, and *r*_*ij*_ represents the rate of emitted pollution produced from burning *i*-th energy by *j*-th user per unit. Forasmuch as the recipient sector can put upon different types of energies such as electricity, fossil fuels, gasoline, and natural gas, the total air pollution that a consumer sector j produces by burning different types of energies is calculated as follows:


(4) $$\begin{array}{l} P_{j}=\overset{m}{\underset{i=1}{\sum }}r_{ij}\chi_{ij}\;,\;for\;1\leq j\leq n, \end{array}$$

where *P*_*j*_ presents the contribution of total pollution generated by user *j* through burning different energies. The objective function of the ERAM model for the entire air pollution should consist of the summation of pollution created by all the customers, i.e., the total air pollution for the operational zone reckons up by the summation of *P*_*j*_ on *i*. Simultaneously, the model is a minimization model, so the objective function can be established as follows:


(5) $$\begin{array}{l} \min\;P_{i,j}=\overset{m}{\underset{i=1}{\sum }}\overset{n}{\underset{j=1}{\sum }}r_{ij}\chi_{ij} \end{array}$$

*Constraints*: All the conditions and limitations of the problem, simulated as a function, make up the constraint functions of our optimization model. These restrictions are composed of supply limitations, air quality expectations, boundaries for air pollution magnitude, lower and upper bounds for demand, and lower and upper bounds for consumption. The tolerance value of each factor fluctuation should be determined.

Since the amount of previous energy sales is assumed as the quantity of supply, each energy can be produced equal to or more than the previous amount. Hence, the constraints of the energy supply would be as follows:


(6) $$\begin{array}{l} \overset{n}{\underset{j=1}{\sum }}\chi_{ij}\geq l_{i},\;i=1,\;2,\ldots ,m,\; \end{array}$$

where *l*_*i*_ is the minimum supply volume of *i*-th energy at different recipients. Accordingly, the condition for the volume of air pollution for each consumer must be equal to or less than the current pollution begets by it. Thus, the constraints can be simulated as follows:


(7) $$\begin{array}{l} \overset{m}{\underset{i=1}{\sum }}r_{ij}\chi_{ij}\leq t_{j},\;j=1,\;2,\;\ldots ,n, \end{array}$$

where *t*_*j*_ is the current air pollution volume emitted by the *j*-th customer through burning different energies.

The feasible solution of χ_*ij*_ should be within the interval of its possible minimum and maximum bounds of the tolerance value based on the situation and limitations. In insomuch, some consumers can only use certain types of energy we need to set a minimum limit for them. Therefore, the simulation function for the specific χ_*ij*_ would be standing equal to or more than the current minimum value, as follows:


(8) $$\begin{array}{l} \chi_{ij}\geq {\chi_{ij}}^{-},\;for\;1\leq i\leq m\;,\;1\leq j\leq n,\\ \;{\chi_{ij}}^{-}>0\;,\chi_{ij}\geq 0. \end{array}$$

On the other hand, due to the limited capacity of some types of energy, a quota should be considered for some specific recipient sectors. Hence, these χ_*ij*_ should endure the simulation function for equal to or less than the maximum value, as follows:


(9) $$\begin{array}{l} \chi_{ij}\;\leq {\chi_{ij}}^{+},\;for\;1\leq i\leq m\;,\;1\leq j\leq n,\;\\ \;{\chi_{ij}}^{+}>0\;,\;\chi_{ij}\geq 0. \end{array}$$

It is worth mentioning that, in Eqs 8 and 9, ${\chi_{ij}}^{-}$ and ${\chi_{ij}}^{+}$ are positive upper and lower bounds of χ_*ij*_. Moreover, the value of χ_*ij*_ must be a non-negative number.

In consequence, the simulation-optimization ERAM model for air pollution simulates in Eq. 2. The optimal solution for χ_*ij*_ obtained through model (2) is the best decision to devote the proper volume of energy to consumers. The optimal value gained from ERAM gives us the lowest amount of air pollution without any changes in current energy usage.

### 3.3. Study area

To illustrate the efficiency of the simulation-optimization ERAM model, the real-world Tehran metropolitan, Iran, was selected. As such, the non-standard Tehran air pollutants were identified based on the WHO Air Quality Index, and the volume of the pollution of non-standard contaminants was determined. The main contributors that make pollution of the non-standard contaminants were investigated, and the energy resources used in Tehran were classified. Eventually, Tehran data for 2018–2019 were used as input for the model.

Tehran is the capital of Iran with a population of around 8.7 million in the city and 15 million multitudes in the metropolitan area of Greater Tehran. In terms of geography, Tehran is located in the north of Iran, surrounded by the Alborz Mountain Chain from the north (18) and Bibi Shahrbanoo Mountain in the southeast. The center of the city is on latitude 35°41′N and longitude 51°26′ E (27) with a total area of more than 700 km^2^.

#### 3.3.1. Tehran's air quality

Similar to other developing countries, Iran has recently encountered air pollution problems. According to a report by the Air Quality Control Company of Tehran (AQCC) (28), the air quality in the metropolis of Tehran is very unhealthy, and most of the AQIs indices are higher than WHO standards. The standard volume guidelines for six air pollution elements mentioned in Section 2.1. are reported by WHO as follows: PM_10_ reported as 20 μg/m^3^ annual mean, PM_2.5_ as 10 μg/m^3^ annual mean, O_3_ as 100 μg/m^3^ 8-h mean, CO as 9 μg/m^3^ annual mean, SO_2_ as 20 μg/m^3^ 24-h mean, and NO_2_ as 40 μg/m^3^ annual mean (29).

The annual mean contribution of Tehran air pollution during 2016–2020 is collected from the AQCC and the Annual Statistics of Tehran Municipality (30) in Table 1.

**Table 1:** Comparison of the annual Tehran air pollution during 2016–2020 with WHO standards.

	**2016–2017**	**2017–2018**	**2018–2019**	**2019–2020**	**WHO standards**
PM_10_	85.8	85.5	72.1	74.2	20
PM_2.5_	31.8	32.4	26.7	29.6	10
O_3_	18.2	19.9	19.7	20.4	100
CO	2.5	2.4	1.8	1.8	9
SO_2_	10.5	7.2	5.3	5.4	20
NO_2_	52.7	53.4	45.4	50.1	40

**Figure 2:**
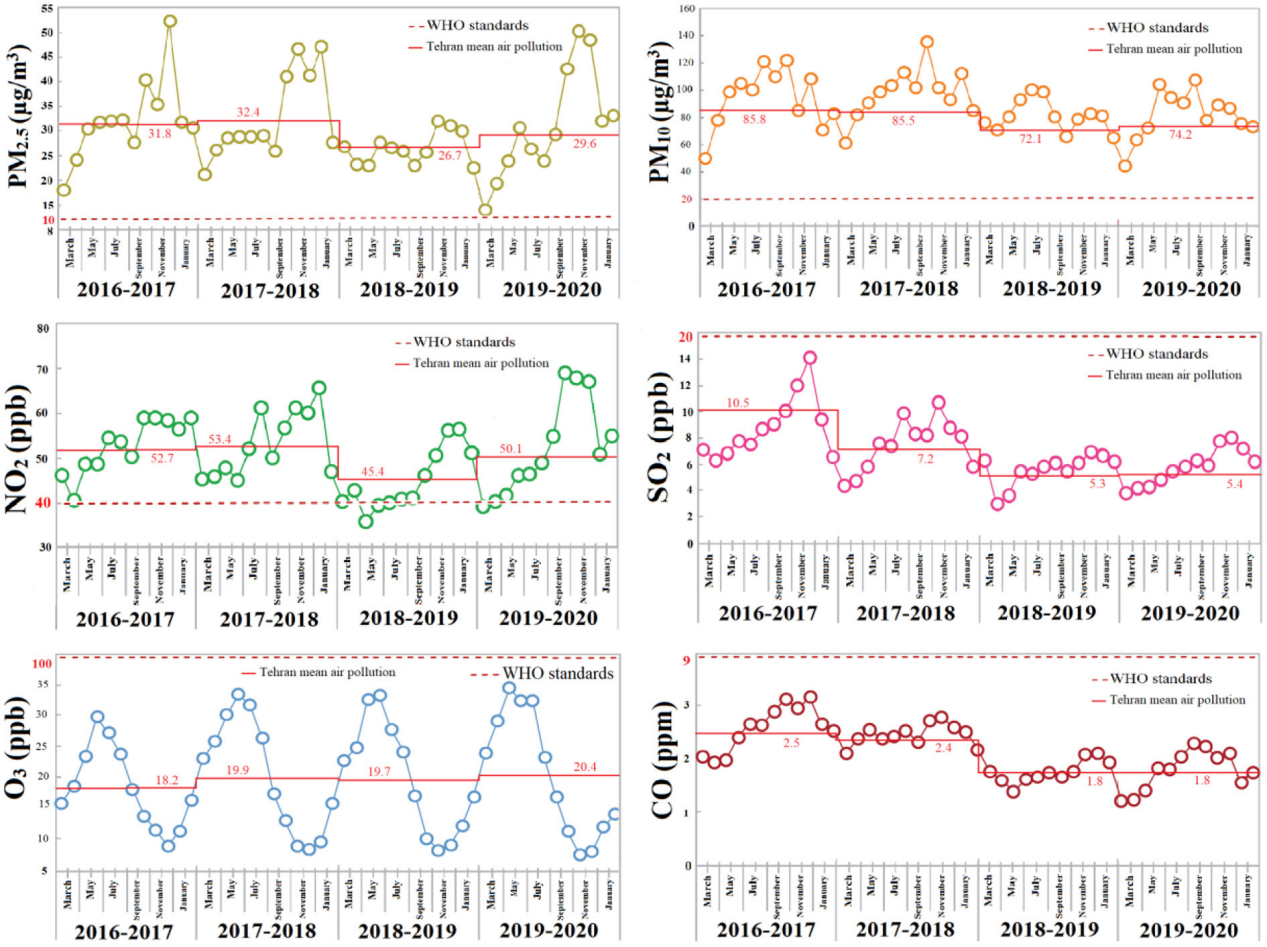
The monthly trend of PM_10_, PM_2.5_, O_3_, NO_2_, CO, and SO_2_ of Tehran for 2016–2020.

*Note*: As the data are presented by the Persian calendar, thus, annual data are in the duration of March of a year to March of the next year.

A glance over the graph of cumulative changes in the concentration of SO_2_, O_3_, and CO pollutants (Figure 2) indicates that government decision-making policies have led the quantity of these contaminants to be lower than WHO standards. The volume of PM_2.5_ and PM_10_ concentrations throughout Tehran implies almost three and four times more than the WHO standards, respectively, which is entirely worrying. The amount of NO_2_ is almost 0.2 times higher than WHO standards, which needs some management.

## 4. Results

In our study area, diesel fuel, gasoline, and compressed natural gas (CNG) have been considered as energy sources. The consumer sectors including motorcycles, cars, heavy-duty vehicles for mobile sources, and industries, household and commercial sources, and energy conversion stations for stationary sources, were identified. We demonstrate that the ERAM model can reduce the air pollution concentration in Tehran by up to 14%. The results confirmed the acceptable accuracy of the model.

### 4.1. Pollution emission by contributors

According to the report of AQCC, the PM_2.5_ pollutant is mainly produced by combustion processes, especially in motor vehicles as well as in the intersection of other pollutants in the air. Furthermore, the most common producers of NO_2_ are vehicles with combustion engines that run on diesel and petrol fuels (31), which directly coincides with the PM emission contributors. Tehran air pollution sources can be categorized into two major parts: mobile sources and stationary sources. The primary sources of PM pollution in Tehran territory are vehicles, which produce nearly 85% of the total pollution and 70% of the PM pollution. The rest of the 30% of PM pollution in Tehran is non-traffic-related emissions, including 20% from energy conversion (including refineries and power plants), 7% from industries, 2% from household and commercial sources, and 1% from gas terminals (18, 32).

#### 4.1.1. Mobile sources

Mobile sources consist of passenger cars, taxis, motorcycles, pickups, private sector buses, minibuses, and trucks. Despite cars being the most abundant vehicle (80% of all vehicles including 90% passenger cars, 8% pickups, and 2% taxis) on the streets of Tehran, they contribute about 3% of the city's mobile PM pollution (2% of the total PM pollution) (33). Although heavy-duty vehicles are the most miniature contributor vehicles (almost 2% of the entire vehicles fleet), they contribute about 85% to mobile PM emissions (60% of the total PM pollution), including private sector buses (35%), Tehran municipal buses (28%), trucks (28%), and minibuses (8%) (18).

Motorcycles are the second largest contributor to PM in Tehran per passenger (18% of total vehicles). Air pollution attributable to motorcycles is about 12% of the total mobile PM emissions amounting (8% of the total PM pollution) to about 1 million petrol motorcycles (34). One of the main reasons for their high contribution to emissions is that motorcycles burn fuel incompletely, which converts to PM under sunlight in the presence of NO_2_.

#### 4.1.2. Stationary sources

Approximately 20% of the Tehran PM pollution is related to energy conversion, consisting of refineries and power plants. Power plant activities are one of the necessities. According to ISNA (35), electricity consumption is directly related to natural gas and oil consumption, considering that almost 94% of the total country's electricity is generated by burning fossil fuels. Various types of power plants in Iran include thermal power plants, combined cycle power plants, nuclear power plants, and hydropower plants (36). Unfortunately, only 0.15% of Tehran's electricity consumption, about 33,856 (Mw/h), is supplied by renewable energy (28). Approximately 7% of the Tehran PM pollution generates by industrial activities, 2% by household and commercial sources, and 1% by gas terminals (32).

**Figure 3:**
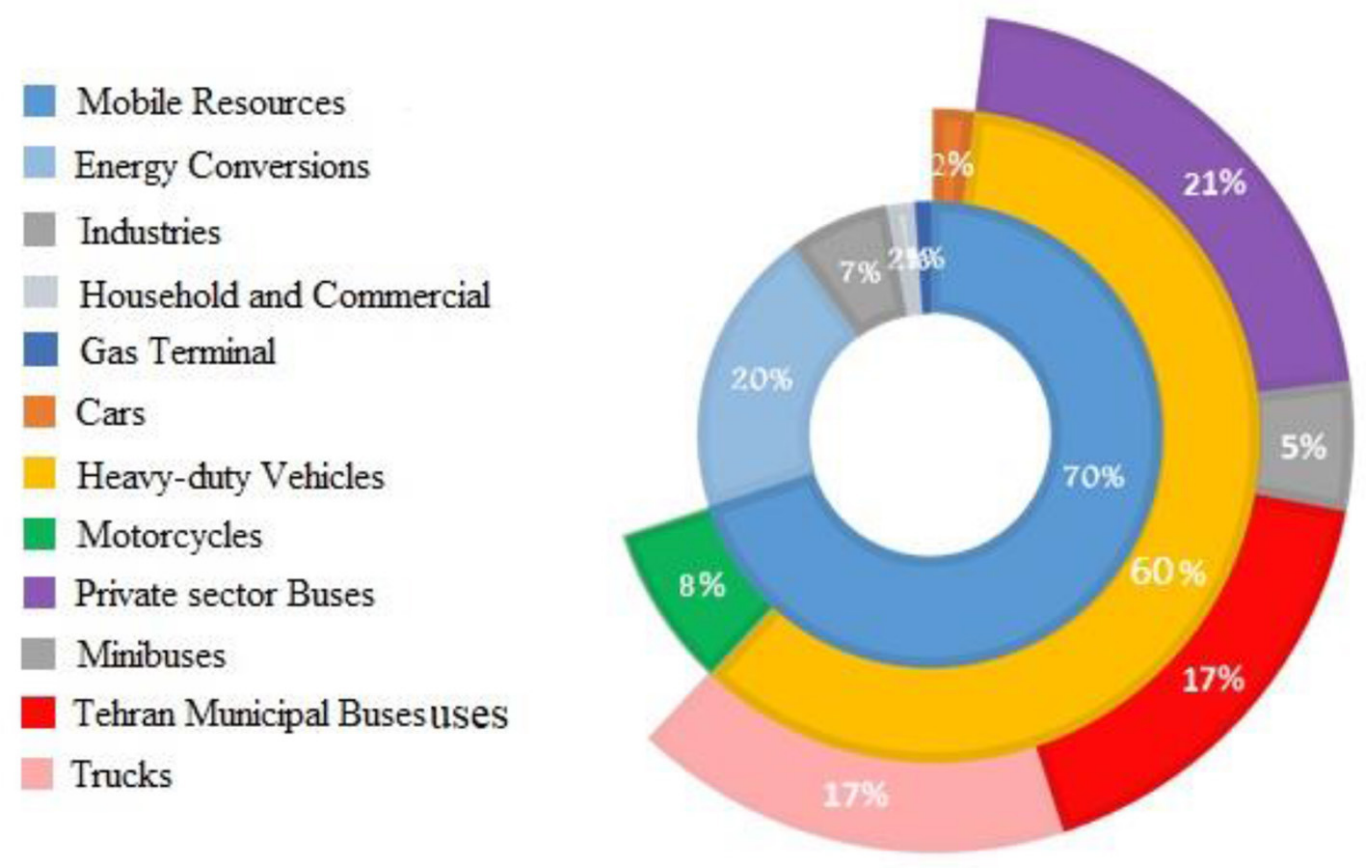
The contribution of the mobile and stationary sources for PM pollution emission in Tehran.

The contribution of the mobile and stationary sources for PM pollution emission in Tehran is shown in Figure 3.

#### 4.1.3. Energy resources

According to the National Iranian Oil Refining and Distributing Company (N.I.O.R.D.C) and Tehran Municipality Statistical annual reports, Tehran fuels sale data were 3,906 million liters of petrol, 1,502 million liters of diesel fuel, and 766.5 million m^3^ of CNG in 2018–2019 (28, 37). In Iran, natural gas is used for daily domestic, commercial, industrial, power plants, and transportation sectors. For heating and cooking purposes, natural gas is the most common and consumed fuel in the household and general service sectors (32). Of this amount, 49% is related to domestic and commercial uses, and the consumption volume for transportation is nearly 4% of the total natural gas consumption (38).

In recent years, the government replaced certain gas, CNG, with petrol for cars (passenger cars, taxis, and pick up) and reduced the aerosols of solid and liquid particles of oil emitted by heavy fuels burning (18). Iran has the largest number of natural gas vehicles in the world, with an estimated 20% of its total vehicle fleet (39). Tehran's taxi fleet includes the largest number of dual-fueled vehicles (33). Only about 8% of the total heavy-duty vehicles are gas-powered (40). Heavy-duty vehicles mostly burn diesel fuel with much higher PM emissions than petrol and CNG. For instance, in idle mode, CNG buses emit 70 mg/h of PM pollution, whereas diesel buses emit 27,030 mg/h (32). It implies that CNG can reduce the PM pollution of diesel fuel by more than 386 times. Thus, CNG is known as a healthy fuel and a proper alternative to diesel fuels to reduce air pollution in Tehran city. This amount in high volume will be substantially effective on air quality. Due to the characteristics of diesel fuel, a large volume of this fuel is consumed in power plants, which replaces the deficit of natural gas delivery to power plants in cold seasons of the year due to the greater demand of the domestic sector for natural gas (38).

Electricity is the most powerful alternative to fossil fuels in reducing air pollution. If the electricity comes from renewable energy sources, e-cars and e-motorcycles are advantageous compared to the hybrid; however, if fossil fuels generate electricity, they do not remain competitive (8) because producing electricity in Tehran causes almost 2.5 times more pollution than current fuel-burn cars and motorcycles pollution in Tehran.

### 4.2. Illustration of the proposed model

*Remark:* Since petroleum products (petrol, diesel fuels, kerosene, mazut, liquefied petroleum gas, aviation fuels, and natural gas) make up an essential part of Iran's economy, in this study, we do not discuss the ability of this country to allocate more quantity of these products to the consumer sectors. We have focused on allocating the proper amount of petroleum products that are currently available and being used. Indeed, the volume of annual average fuel sales, which is defined as customer demand, is assumed as the quantity of supply.

*Step 1:* In Section 3.3.1., the trend of decreasing and increasing for six major air pollution contaminants of Tehran city was identified and compared with the WHO standards. Values above the bars were noticed. Data were collected according to the non-standard pollutants. In Section 4.1., the most significant air pollution emission factors were detected, and the ratio of each factor's contribution to air pollution was surveyed precisely. In this study, terminal gas pollution is not considered an air pollution contributor because it makes up just 1% of the pollution (Figure 3), which is related to fuel burning and will automatically reduce forasmuch through fuel allocation management between recipients. In Section 4.2., the amount of fuel supply was taken from the 2018–2019 petroleum diversity products sales over Tehran. As more than 94% of Tehran's electricity is produced by burning fossil fuels, therefore, electricity cannot be a proper alternative energy to fuel-powered devices. For this issue, in this research, petroleum products consisting of petrol, diesel fuel, and CNG are considered the only resources for this city's consumption of energy required.

Moreover, the impact rate of each contributor on making air pollution is non-identical based on the type of user, the type of energy-power burning, and the voluminosity of energy burns by each user sector, i.e., the proportion of each consumer in generating ambient pollution by burning different fuels per unit (*r*_*ij*_) is different individually. The required data for creating the ERAM model are collected in Table 2 (13, 21, 28, 32).

**Table 2:** Tehran data.

		**1 Motorcycle**	**2 Car**	**3 Heavy-duty vehicle**	**4 Energy conversion**	**5 Industry**	**6 Household and commercial**	**Total supply**
	** 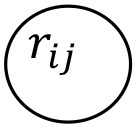 **							
1	Petrol	0.47	0.11			0.44		3,906
2	Diesel fuel			27	2.54	1.5		1,502
3	CNG		0.04	0.07	0.25	0.02	0.55	766.5
	*P*_j_ (ton/year)	8%	2%	60%	20%	7%	2%	**10,967**
		863	209	6,756	2,161	763	215	

The bold values indicate the Tehran's total air pollution 2018–2019.

*Step 2:* ERAM model: From Section 3.2., the ERAM model is built in two parts, objective functions and constraints. Here, we simulate the ERAM model based on the Tehran situation to calculate the best assignment of fuels to the consumers systematically.

*Objective function:* The objective function of the problem is concerned with the air pollution that is expected to be reduced. Thus, the objective function is a minimization of the total PM pollution problem in the metropolis of Tehran consisting of the summation of all PM pollutant contributors multiplied by the ratio of their impact factor per unit (Eq. 5).


(10) $$\begin{array}{c} \min\;\overset{m}{\underset{i=1}{\sum }}\overset{n}{\underset{j=1}{\sum }}r_{ij}\chi_{ij}=0.47\;\chi_{11}+0.11\;\chi_{12}\\ +0.04\;\chi_{32}+27\;\chi_{23}+0.07\;\chi_{33}+2.54\;\chi_{24}\\ +\;0.25\;\chi_{34}+0.44\;\chi_{15}+1.5\;\chi_{25}\\ \;+0.02\;\chi_{35}+0.55\;\chi_{36}\; \end{array}$$

*Constraints:* As mentioned, the capability of donor reservoir storage, which is supposed not to exceed the 2018–2019 sales demand fuels, is considered the estimated amount for supply fuels. According to Table 2, we can have the following constraints on fuel energy sources, petrol, diesel fuel, and CNG, by exerting the data into Eq. 6.


(11) $$\begin{array}{c} \chi_{11}+\chi_{12}+\chi_{13}\geq 3906,\\ \;\chi_{23}+\chi_{24}+\chi_{25}\geq 1502,\\ \chi_{32}+\chi_{33}+\chi_{34}+\chi_{35}+\chi_{36}\geq 766.5. \end{array}$$

The second set of constraints is relevant to the current air pollution. For simulating the constraints through Eq. 7, *r*_*ij*_ and the content of current air pollution existing as shown in Table 2 are needed. The six consumers create the six constraints as follows:


(12) $$\begin{array}{c} 0.47\;\chi_{11}\leq 863,\\ 0.11\;\chi_{12}+0.04\;\chi_{32}\leq 209,\\ 27\;\chi_{23}+0.07\;\chi_{33}\leq 6756,\\ 2.4\;\chi_{24}+0.25\;\chi_{34}\leq 2161,\\ 0.44\;\chi_{15}+1.5\;\chi_{25}+0.02\;\chi_{35}\leq 763,\\ 0.55\;\chi_{36}\leq 215. \end{array}$$

There exist some constraints related to the upper and lower amount of fuel supply and consumer demand. The director of Thermal Electricity Support reminded us that over 90% of the fuel required by power plants is supplied with natural gas annually in the hot seasons; however, in cold seasons due to the greater demand of the domestic sector for natural gas, the consumption of power plants turns to the use of diesel fuel (41). Hence, we cannot devote more gas quota to this segment in cold months. For this issue, the minimum consumption of diesel fuel should be chosen as the lower-bound condition for the energy conversion sector. In addition, for the same reason, restriction on domestic sector CNG consumption is simulated through Eq. 8, as follows:


(13) $$\begin{array}{c} \chi_{24}\;\geq 72.5,\\ \chi_{36}\;\geq 376,\\ \chi_{ij}\geq 0,\;fori=1,\;\ldots ,3,\;j=1,\;\ldots ,\;6\;. \end{array}$$

In practice, Eq. 10 as the objective function of our model and Eqs 11, 12, and 13 as the constraints create the following ERAM model to reduce the Tehran detrimental PM pollution.


(14) $$\begin{array}{c} min\;0.47\;\chi_{11}+0.11\;\chi_{12}+0.04\;\chi_{32}+27\;\chi_{23}+0.07\;\chi_{33}\\ +2.54\;\chi_{24}+0.25\;\chi_{34}+0.44\;\chi_{15}+1.5\;\chi_{25}\\ +0.02\;\chi_{35}+0.55\;\chi_{36}\\ s.t.\;\chi_{11}+\chi_{12}+\chi_{13}\geq 3906,\\ \chi_{23}+\chi_{24}+\chi_{25}\geq 1502,\\ \chi_{32}+\chi_{33}+\chi_{34}+\chi_{35}+\chi_{36}\geq 766.5,\\ 0.47\;\chi_{11}\leq 863,\\ 0.11\;\chi_{12}+0.04\;\chi_{32}\leq 209,\\ 27\;\chi_{23}+0.07\;\chi_{33}\leq 6756,\\ 2.4\;\chi_{24}+0.25\;\chi_{34}\leq 2161,\\ 0.44\;\chi_{15}+1.5\;\chi_{25}+0.02\;\chi_{35}\leq 763,\\ 0.55\;\chi_{36}\leq 215,\\ \chi_{24}\;\geq 72.5,\\ \chi_{36}\;\geq 376,\\ \chi_{ij}\geq 0,\;fori=1,\;\ldots ,3,\;j=1,\;\ldots ,\;6\;. \end{array}$$

**Figure 4:**
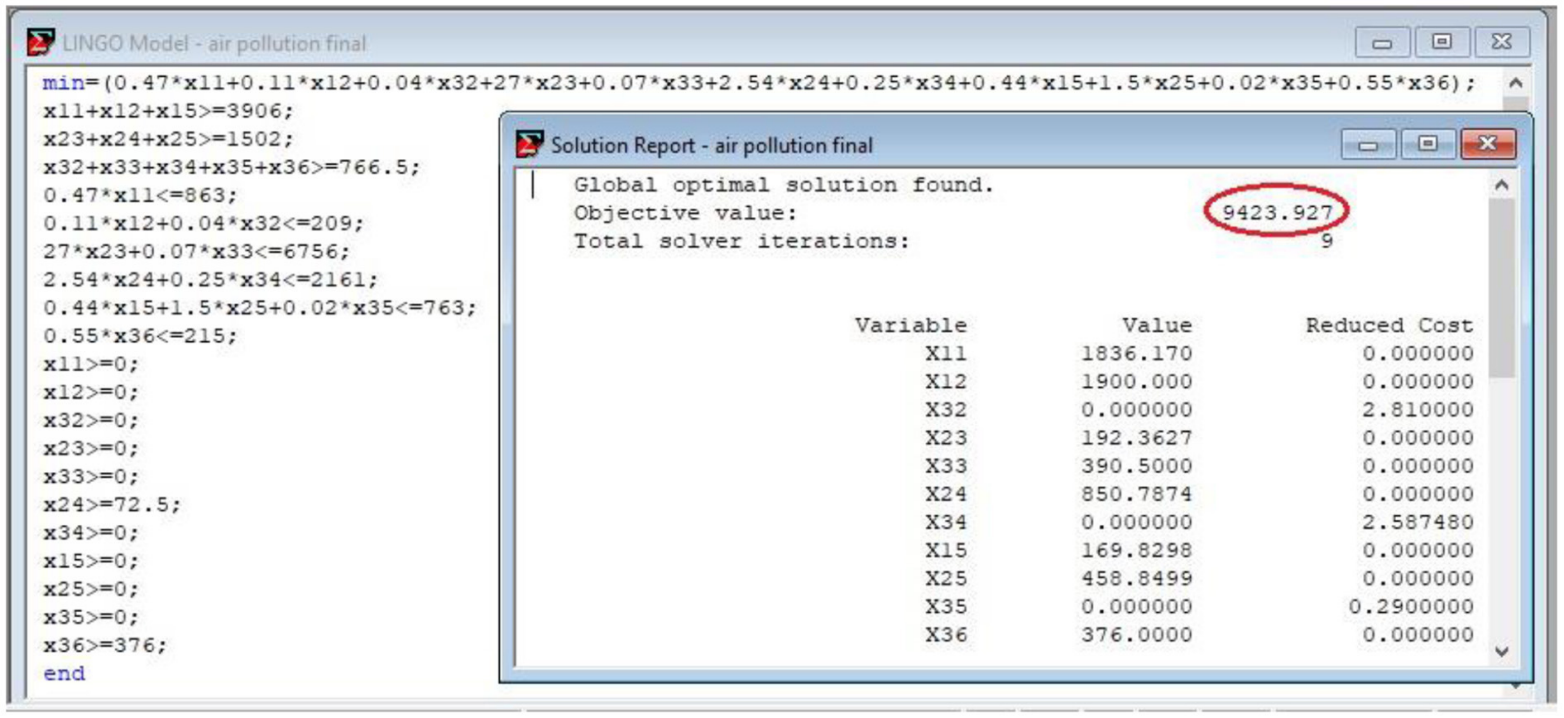
Highest air pollution reduction of Tehran.

As for the output of the ERAM derived in Figure 4, the energy allocation management has been in the proper way to decrease the amount of diesel fuel consumption pattern for heavy-duty vehicles, as it is the most air pollution producer in Tehran. Beyond this, the volume of demand fuel for heavy-duty vehicles sector is supported by CNG instead of declining air pollution substantially. The excellence of fit for the evaluation and validation of the ERAM model is well illustrated through explicit-decreasing Tehran air pollution by up to 14.07% from 10,967 to 9,424 (ton/year). Meanwhile, the assessment of model performance in the study area is in the satisfactory range. It is noteworthy that this reduction in air pollution was without intervening with fuel consumption amount or imposing any restrictions on consumers.

## 5. Comparison analysis

In this section, the final output of the proposed method is compared to the previous studies to illustrate and investigate the efficiency and priority of the ERAM model.

For reducing the PM_10_ pollution caused by traffic emissions, some transit bans were applied for heavy-duty vehicles in Munich (42). For this issue, a semi-parametric regression model was proposed for measuring the effect of these bans on PM_10_ mass concentrations. The results showed a PM_10_ pollution reduction of 6.8% in winter to 13% in summer in traffic monitoring sites. It is evident that, even by implementing bans on heavy-duty vehicles, the amount of pollution reduction was less than the ERAM model. A study on 83 cities in china showed that promoting the Urban Traffic Infrastructure Investment (UTII) policy can reduce air pollution. In this research, the fixed-effect model and dynamic panel data model were used. Estimation of long-time policy indicated the air pollution reduction to 5% (43).

In (44), the authors assessed the effectiveness of vehicles' lower speed in reducing the value of PM_10_, PM_1_, Black Smoke, and NO_2_ pollution, related to the traffic emission on a highway in Amsterdam. A linear multivariate regression model was used to measure the effect of traffic contribution. The results showed a 7.4% reduction in PM_10_ and a 2.4% reduction in NO_2_ contaminants. A previous study (45) states that the policy of the government to reduce air pollution was applying a variable speed limit system on some city access routes. To measure the impact of those policies on NO_2_ and PM_10_ pollutants reduction, a differences-in-differences method was used. Outcomes implied a 7.7%−17.1% reduction in NO_2_ and 14.5%−17.3% in PM_10_ in Barcelona. Although their policy could reduce air pollution more than the ERAM model, without the applied limitations, they could not access this volume of reduction. In Yap and Garcia's study (46), a policy for reducing wood burning, mostly during wintertime, was established as a Rule in San Joaquin Valley Air Basin (SJVAB), California. This policy was adopted to reduce the residential wood-burning fireplaces' CO and PM concentration emissions. General linear-mixed models were used to compare the levels of PM pollution. The results implied a 12% reduction in PM_2.5_ and 8% in coarse particles all over the SJVAB, although these values were 11 and 15% for PM_2.5_ and 7 and 11% for coarse particles in rural and urban areas, respectively. Less wood burning in heater fireplaces in cold weather is a big limitation that makes some difficulties in lifestyle for residents.

## 6. Discussion

In this study, a novel mathematical model has been proposed to cope with the air pollution problem, denoted as the ERAM model. Methodologically, an integrated simulation-optimization ERAM model proved to be a practical and appropriate tool to redact strategies for air pollution reduction, which is precisely simulated under a decision-making tolerance value and limitations of the study area, to optimize the energy resource allocation between different recipient sectors. This optimization can open an opportunity for decision-makers to air pollution mitigation as much as possible.

The real-world case study for evaluating the influence and performance of the compatible proposed method was Tehran. The ERAM is modeled based on the limitation on different types of energy resources and the restrictions of production and distribution, which are all modeled in the constraints part of the ERAM model. The strength of this method is that we do not go through changing the limitations. Any changes need cost, time, and government or residence corporation, such as the need for citizen participation, alternative fuels, or any policies on a fuel-burn style that we can see in previous studies. Since the long-term implementation of a restriction will be difficult for the residents of that area, we proposed the ERAM model not to make any surplus limitations for consumers.

Higher exposure to air pollution such as PM_2.5_ is associated with higher mortality from cardiovascular and respiratory diseases (47). Further, analysis of documents on the reduction of life expectancy in the case of transboundary pollution can show the impact of the globalization of pollution on people's lives. The results indicated that air pollution increasing causes 1.03 million premature deaths and reduced the global average life expectancy by 0.23 years, which is almost 84 days of human life (48). Then, it is a very considerable issue for human health to reduce air pollution. The ERAM model can be an applicable and efficient model to reduce air pollution and increase life expectancy as well as reduce the mortality from diseases caused by air pollution, as it does not need any policy or changes in citizens' routine fuel consumption.

In this study, we planned to collect Tehran's data for 5 consecutive years so that we could check the effect of using the proposed method on reducing air pollution. We also wanted to see how much this reduction in contamination can increase life expectancy. Unfortunately, the required data were not available for 5 years.

## 7. Conclusion and future works

This study illustrated that high air pollution levels could vanish by making a correct decision on allocating different energies to user sectors. The results showed that the ERAM model could impressively reduce Tehran's air pollution by up to 14% without making any changes to the fuel-burn style, extra costs and time, or applying some policies to people's lifestyles. The proposed model in this method is designed based on the current situation and the facilities of the study area. As it is mentioned in Section 4.3, we focused on modifying the current situation, not changing it.

The ERAM model analysis indicated that logistical air pollution could highly decrease when a slight change in assigning energy occurs. This can lead the decision-makers to changes in the quantity, intensity, and velocity of air pollutant diffusions. The ERAM model is simulated based on an energy-consuming assignment, showing that optimal allocating of various fuels to consumers can significantly reduce air pollution. An actual case study, Tehran, is chosen to evaluate the effectiveness of the proposed ERAM model.

For future work, we intend to observe the effect of a 14% air pollution reduction on the number of patients with polluted air diseases and measure the increasing life expectancy, by applying the ERAM model to several counties and comparing the results.

## Data availability statement

The datasets presented in this study can be found in online repositories. The names of the repository/repositories and accession number(s) can be found in the article/supplementary material.

## Author contributions

AK conducted the conceptualization, methodology, software, formal analysis, resources, data curation, investigation, and writing the original draft. LL reviewed the manuscript. All authors contributed to the article and approved the submitted version.
